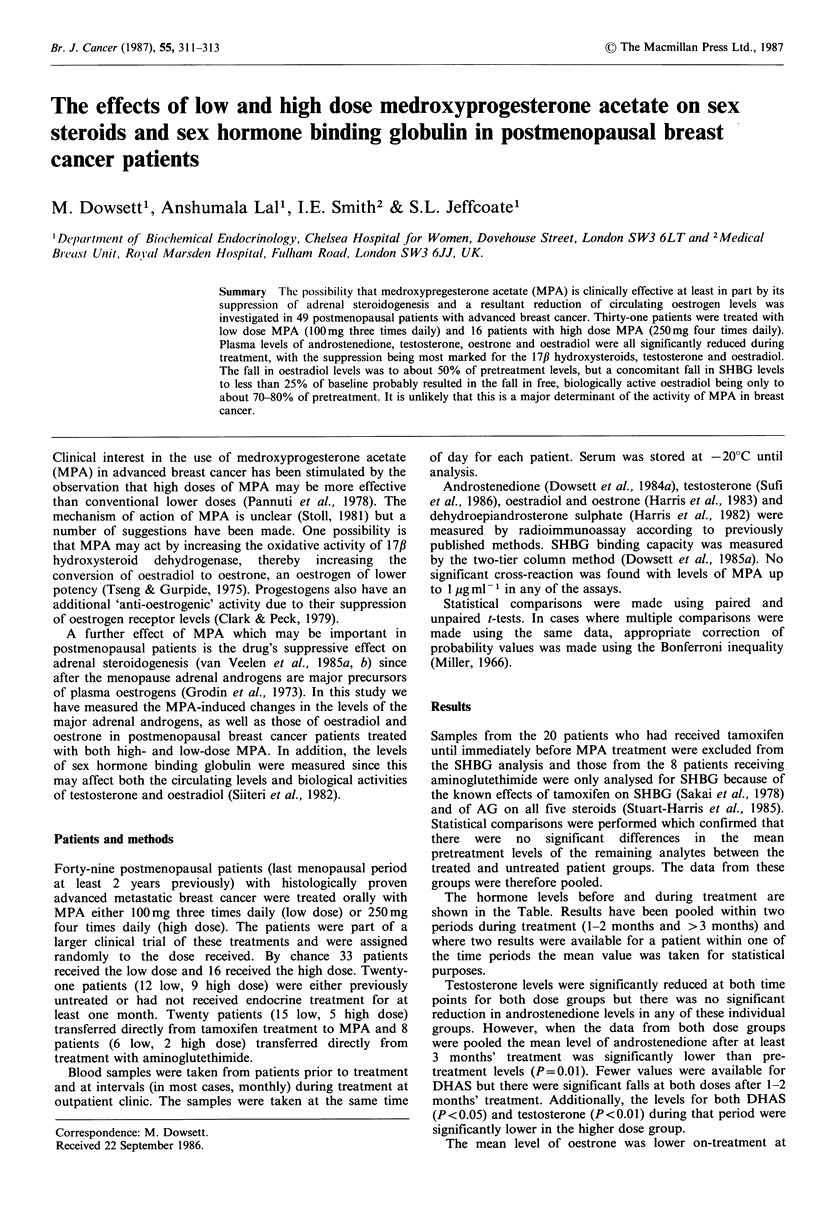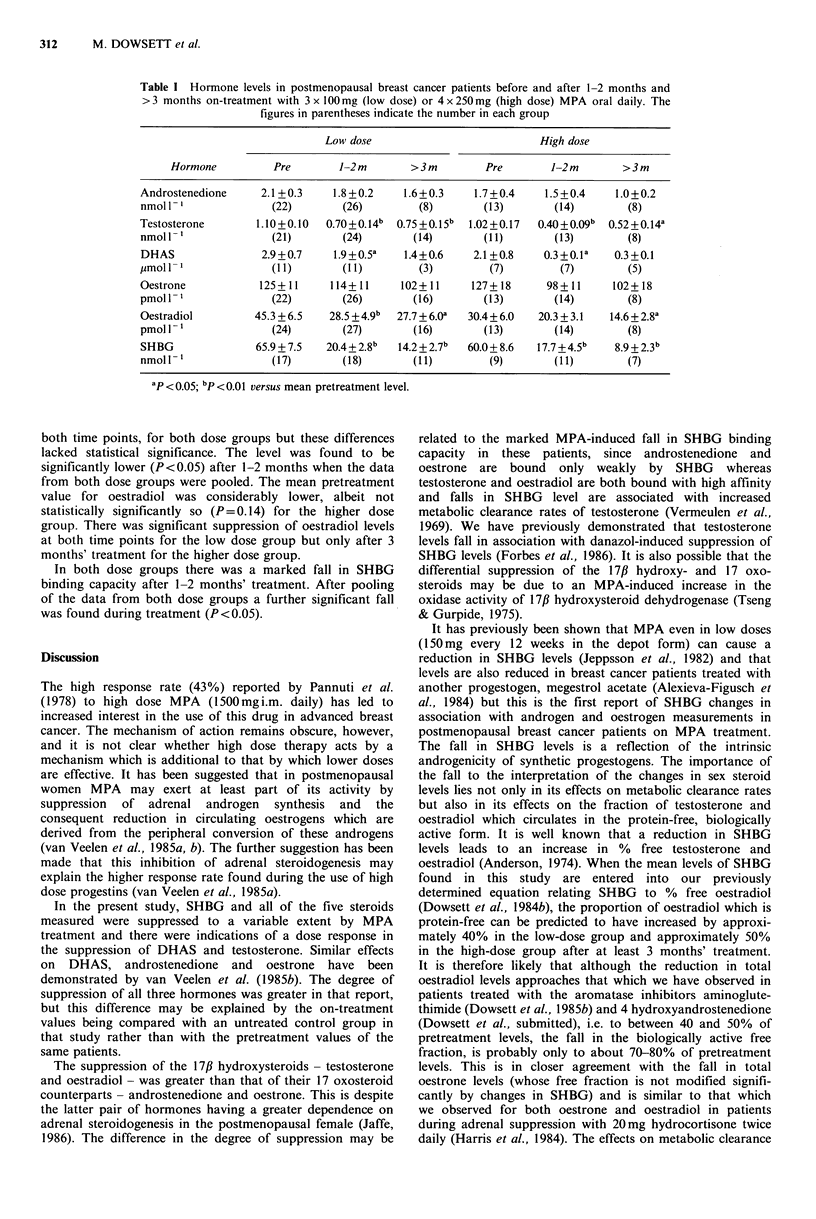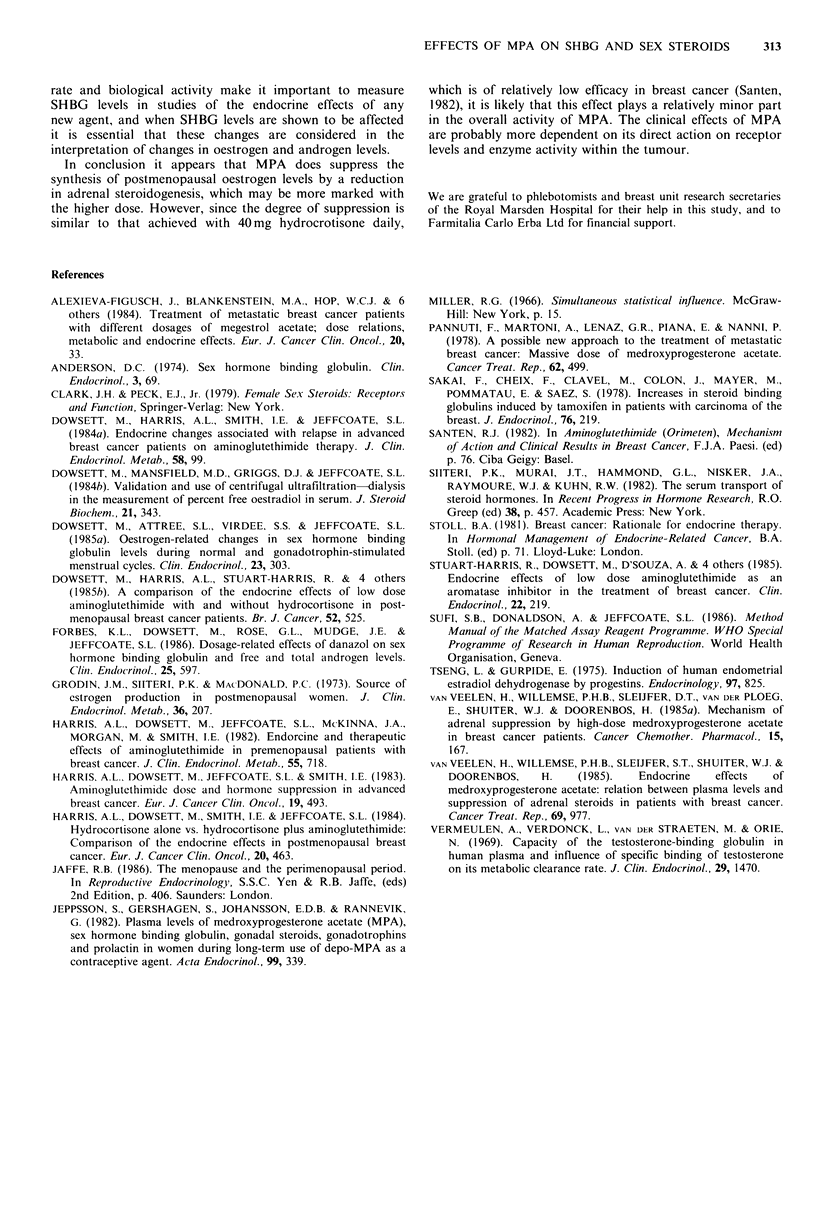# The effects of low and high dose medroxyprogesterone acetate on sex steroids and sex hormone binding globulin in postmenopausal breast cancer patients.

**DOI:** 10.1038/bjc.1987.61

**Published:** 1987-03

**Authors:** M. Dowsett, A. Lal, I. E. Smith, S. L. Jeffcoate

## Abstract

The possibility that medroxyprogesterone acetate (MPA) is clinically effective at least in part by its suppression of adrenal steroidogenesis and a resultant reduction of circulating oestrogen levels was investigated in 49 postmenopausal patients with advanced breast cancer. Thirty-one patients were treated with low dose MPA (100 mg three times daily) and 16 patients with high dose MPA (250 mg four times daily). Plasma levels of androstenedione, testosterone, oestrone and oestradiol were all significantly reduced during treatment, with the suppression being most marked for the 17 beta hydroxysteroids, testosterone and oestradiol. The fall in oestradiol levels was to about 50% of pretreatment levels, but a concomitant fall in SHBG levels to less than 25% of baseline probably resulted in the fall in free, biologically active oestradiol being only to about 70-80% of pretreatment. It is unlikely that this is a major determinant of the activity of MPA in breast cancer.


 © The Macmillan Press Ltd., 1987

### The effects of low and high dose medroxyprogesterone acetate on sex steroids and sex hormone binding globulin in postmenopausal breast cancer patients

M. Dowsett[1], Anshumala Lal[1], I.E. Smith[2] & S.L. Jeffcoate[1]

[1]*Department of Biochemical Endocrinology, Chelsea Hospital for Women, Dovehouse Street, London SW3 6LT and* [2]*Medical Breast Unit, Royal Marsden Hospital, Fulham Road, London SW3 6JJ, UK.*

**Summary** The possibility that medroxypregesterone acetate (MPA) is clinically effective at least in part by its suppression of adrenal steroidogenesis and a resultant reduction of circulating oestrogen levels was investigated in 49 postmenopausal patients with advanced breast cancer. Thirty-one patients were treated with low dose MPA (100 mg three times daily) and 16 patients with high dose MPA (250 mg four times daily). Plasma levels of androstenedione, testosterone, oestrone and oestradiol were all significantly reduced during treatment, with the suppression being most marked for the 17$\beta$ hydroxysteroids, testosterone and oestradiol. The fall in oestradiol levels was to about 50% of pretreatment levels, but a concomitant fall in SHBG levels to less than 25% of baseline probably resulted in the fall in free, biologically active oestradiol being only to about 70–80% of pretreatment. It is unlikely that this is a major determinant of the activity of MPA in breast cancer.

Clinical interest in the use of medroxyprogesterone acetate (MPA) in advanced breast cancer has been stimulated by the observation that high doses of MPA may be more effective than conventional lower doses (Pannuti *et al.*, 1978). The mechanism of action of MPA is unclear (Stoll, 1981) but a number of suggestions have been made. One possibility is that MPA may act by increasing the oxidative activity of 17$\beta$ hydroxysteroid dehydrogenase, thereby increasing the conversion of oestradiol to oestrone, an oestrogen of lower potency (Tseng & Gurpide, 1975). Progestogens also have an additional 'anti-oestrogenic' activity due to their suppression of oestrogen receptor levels (Clark & Peck, 1979).

A further effect of MPA which may be important in postmenopausal patients is the drug's suppressive effect on adrenal steroidogenesis (van Veelen *et al.*, 1985*a*, *b*) since after the menopause adrenal androgens are major precursors of plasma oestrogens (Grodin *et al.*, 1973). In this study we have measured the MPA-induced changes in the levels of the major adrenal androgens, as well as those of oestradiol and oestrone in postmenopausal breast cancer patients treated with both high- and low-dose MPA. In addition, the levels of sex hormone binding globulin were measured since this may affect both the circulating levels and biological activities of testosterone and oestradiol (Siiteri *et al.*, 1982).

### Patients and methods

Forty-nine postmenopausal patients (last menopausal period at least 2 years previously) with histologically proven advanced metastatic breast cancer were treated orally with MPA either 100 mg three times daily (low dose) or 250 mg four times daily (high dose). The patients were part of a larger clinical trial of these treatments and were assigned randomly to the dose received. By chance 33 patients received the low dose and 16 received the high dose. Twenty-one patients (12 low, 9 high dose) were either previously untreated or had not received endocrine treatment for at least one month. Twenty patients (15 low, 5 high dose) transferred directly from tamoxifen treatment to MPA and 8 patients (6 low, 2 high dose) transferred directly from treatment with aminoglutethimide.

Blood samples were taken from patients prior to treatment and at intervals (in most cases, monthly) during treatment at outpatient clinic. The samples were taken at the same time of day for each patient. Serum was stored at $-20$°C until analysis.

Androstenedione (Dowsett *et al.*, 1984*a*), testosterone (Sufi *et al.*, 1986), oestradiol and oestrone (Harris *et al.*, 1983) and dehydroepiandrosterone sulphate (Harris *et al.*, 1982) were measured by radioimmunoassay according to previously published methods. SHBG binding capacity was measured by the two-tier column method (Dowsett *et al.*, 1985*a*). No significant cross-reaction was found with levels of MPA up to 1 $\mu$g ml$^{-1}$ in any of the assays.

Statistical comparisons were made using paired and unpaired *t*-tests. In cases where multiple comparisons were made using the same data, appropriate correction of probability values was made using the Bonferroni inequality (Miller, 1966).

### Results

Samples from the 20 patients who had received tamoxifen until immediately before MPA treatment were excluded from the SHBG analysis and those from the 8 patients receiving aminoglutethimide were only analysed for SHBG because of the known effects of tamoxifen on SHBG (Sakai *et al.*, 1978) and of AG on all five steroids (Stuart-Harris *et al.*, 1985). Statistical comparisons were performed which confirmed that there were no significant differences in the mean pretreatment levels of the remaining analytes between the treated and untreated patient groups. The data from these groups were therefore pooled.

The hormone levels before and during treatment are shown in the Table. Results have been pooled within two periods during treatment (1–2 months and >3 months) and where two results were available for a patient within one of the time periods the mean value was taken for statistical purposes.

Testosterone levels were significantly reduced at both time points for both dose groups but there was no significant reduction in androstenedione levels in any of these individual groups. However, when the data from both dose groups were pooled the mean level of androstenedione after at least 3 months' treatment was significantly lower than pre-treatment levels ($P=0.01$). Fewer values were available for DHAS but there were significant falls at both doses after 1–2 months' treatment. Additionally, the levels for both DHAS ($P<0.05$) and testosterone ($P<0.01$) during that period were significantly lower in the higher dose group.

The mean level of oestrone was lower on-treatment at

Correspondence: M. Dowsett.
Received 22 September 1986.

**Table I** Hormone levels in postmenopausal breast cancer patients before and after 1–2 months and >3 months on-treatment with 3×100mg (low dose) or 4×250mg (high dose) MPA oral daily. The figures in parentheses indicate the number in each group

| | Low dose | | | High dose | | |
|---|---|---|---|---|---|---|
| *Hormone* | *Pre* | *1–2m* | *>3m* | *Pre* | *1–2m* | *>3m* |
| Androstenedione $nmol\,l^{-1}$ | 2.1±0.3 (22) | 1.8±0.2 (26) | 1.6±0.3 (8) | 1.7±0.4 (13) | 1.5±0.4 (14) | 1.0±0.2 (8) |
| Testosterone $nmol\,l^{-1}$ | 1.10±0.10 (21) | 0.70±0.14[b] (24) | 0.75±0.15[b] (14) | 1.02±0.17 (11) | 0.40±0.09[b] (13) | 0.52±0.14[a] (8) |
| DHAS $\mu mol\,l^{-1}$ | 2.9±0.7 (11) | 1.9±0.5[a] (11) | 1.4±0.6 (3) | 2.1±0.8 (7) | 0.3±0.1[a] (7) | 0.3±0.1 (5) |
| Oestrone $pmol\,l^{-1}$ | 125±11 (22) | 114±11 (26) | 102±11 (16) | 127±18 (13) | 98±11 (14) | 102±18 (8) |
| Oestradiol $pmol\,l^{-1}$ | 45.3±6.5 (24) | 28.5±4.9[b] (27) | 27.7±6.0[a] (16) | 30.4±6.0 (13) | 20.3±3.1 (14) | 14.6±2.8[a] (8) |
| SHBG $nmol\,l^{-1}$ | 65.9±7.5 (17) | 20.4±2.8[b] (18) | 14.2±2.7[b] (11) | 60.0±8.6 (9) | 17.7±4.5[b] (11) | 8.9±2.3[b] (7) |

[a]$P<0.05$; [b]$P<0.01$ *versus* mean pretreatment level.

both time points, for both dose groups but these differences lacked statistical significance. The level was found to be significantly lower ($P<0.05$) after 1–2 months when the data from both dose groups were pooled. The mean pretreatment value for oestradiol was considerably lower, albeit not statistically significantly so ($P=0.14$) for the higher dose group. There was significant suppression of oestradiol levels at both time points for the low dose group but only after 3 months' treatment for the higher dose group.

In both dose groups there was a marked fall in SHBG binding capacity after 1–2 months' treatment. After pooling of the data from both dose groups a further significant fall was found during treatment ($P<0.05$).

### Discussion

The high response rate (43%) reported by Pannuti *et al.* (1978) to high dose MPA (1500mgi.m. daily) has led to increased interest in the use of this drug in advanced breast cancer. The mechanism of action remains obscure, however, and it is not clear whether high dose therapy acts by a mechanism which is additional to that by which lower doses are effective. It has been suggested that in postmenopausal women MPA may exert at least part of its activity by suppression of adrenal androgen synthesis and the consequent reduction in circulating oestrogens which are derived from the peripheral conversion of these androgens (van Veelen *et al.*, 1985*a*, *b*). The further suggestion has been made that this inhibition of adrenal steroidogenesis may explain the higher response rate found during the use of high dose progestins (van Veelen *et al.*, 1985*a*).

In the present study, SHBG and all of the five steroids measured were suppressed to a variable extent by MPA treatment and there were indications of a dose response in the suppression of DHAS and testosterone. Similar effects on DHAS, androstenedione and oestrone have been demonstrated by van Veelen *et al.* (1985*b*). The degree of suppression of all three hormones was greater in that report, but this difference may be explained by the on-treatment values being compared with an untreated control group in that study rather than with the pretreatment values of the same patients.

The suppression of the 17β hydroxysteroids – testosterone and oestradiol – was greater than that of their 17 oxosteroid counterparts – androstenedione and oestrone. This is despite the latter pair of hormones having a greater dependence on adrenal steroidogenesis in the postmenopausal female (Jaffe, 1986). The difference in the degree of suppression may be related to the marked MPA-induced fall in SHBG binding capacity in these patients, since androstenedione and oestrone are bound only weakly by SHBG whereas testosterone and oestradiol are both bound with high affinity and falls in SHBG level are associated with increased metabolic clearance rates of testosterone (Vermeulen *et al.*, 1969). We have previously demonstrated that testosterone levels fall in association with danazol-induced suppression of SHBG levels (Forbes *et al.*, 1986). It is also possible that the differential suppression of the 17β hydroxy- and 17 oxo-steroids may be due to an MPA-induced increase in the oxidase activity of 17β hydroxysteroid dehydrogenase (Tseng & Gurpide, 1975).

It has previously been shown that MPA even in low doses (150mg every 12 weeks in the depot form) can cause a reduction in SHBG levels (Jeppsson *et al.*, 1982) and that levels are also reduced in breast cancer patients treated with another progestogen, megestrol acetate (Alexieva-Figusch *et al.*, 1984) but this is the first report of SHBG changes in association with androgen and oestrogen measurements in postmenopausal breast cancer patients on MPA treatment. The fall in SHBG levels is a reflection of the intrinsic androgenicity of synthetic progestogens. The importance of the fall to the interpretation of the changes in sex steroid levels lies not only in its effects on metabolic clearance rates but also in its effects on the fraction of testosterone and oestradiol which circulates in the protein-free, biologically active form. It is well known that a reduction in SHBG levels leads to an increase in % free testosterone and oestradiol (Anderson, 1974). When the mean levels of SHBG found in this study are entered into our previously determined equation relating SHBG to % free oestradiol (Dowsett *et al.*, 1984*b*), the proportion of oestradiol which is protein-free can be predicted to have increased by approximately 40% in the low-dose group and approximately 50% in the high-dose group after at least 3 months' treatment. It is therefore likely that although the reduction in total oestradiol levels approaches that which we have observed in patients treated with the aromatase inhibitors aminoglutethimide (Dowsett *et al.*, 1985*b*) and 4 hydroxyandrostenedione (Dowsett *et al.*, submitted), i.e. to between 40 and 50% of pretreatment levels, the fall in the biologically active free fraction, is probably only to about 70–80% of pretreatment levels. This is in closer agreement with the fall in total oestrone levels (whose free fraction is not modified significantly by changes in SHBG) and is similar to that which we observed for both oestrone and oestradiol in patients during adrenal suppression with 20mg hydrocortisone twice daily (Harris *et al.*, 1984). The effects on metabolic clearance rate and biological activity make it important to measure SHBG levels in studies of the endocrine effects of any new agent, and when SHBG levels are shown to be affected it is essential that these changes are considered in the interpretation of changes in oestrogen and androgen levels.

In conclusion it appears that MPA does suppress the synthesis of postmenopausal oestrogen levels by a reduction in adrenal steroidogenesis, which may be more marked with the higher dose. However, since the degree of suppression is similar to that achieved with 40 mg hydrocrotisone daily, which is of relatively low efficacy in breast cancer (Santen, 1982), it is likely that this effect plays a relatively minor part in the overall activity of MPA. The clinical effects of MPA are probably more dependent on its direct action on receptor levels and enzyme activity within the tumour.

We are grateful to phlebotomists and breast unit research secretaries of the Royal Marsden Hospital for their help in this study, and to Farmitalia Carlo Erba Ltd for financial support.

### References

ALEXIEVA-FIGUSCH, J., BLANKENSTEIN, M.A., HOP, W.C.J. & 6 others (1984). Treatment of metastatic breast cancer patients with different dosages of megestrol acetate; dose relations, metabolic and endocrine effects. *Eur. J. Cancer Clin. Oncol.*, **20**, 33.

ANDERSON, D.C. (1974). Sex hormone binding globulin. *Clin. Endocrinol.*, **3**, 69.

CLARK, J.H. & PECK, E.J., Jr. (1979). *Female Sex Steroids: Receptors and Function*, Springer-Verlag: New York.

DOWSETT, M., HARRIS, A.L., SMITH, I.E. & JEFFCOATE, S.L. (1984*a*). Endocrine changes associated with relapse in advanced breast cancer patients on aminoglutethimide therapy. *J. Clin. Endocrinol. Metab.*, **58**, 99.

DOWSETT, M., MANSFIELD, M.D., GRIGGS, D.J. & JEFFCOATE, S.L. (1984*b*). Validation and use of centrifugal ultrafiltration—dialysis in the measurement of percent free oestradiol in serum. *J. Steroid Biochem.*, **21**, 343.

DOWSETT, M., ATTREE, S.L., VIRDEE, S.S. & JEFFCOATE, S.L. (1985*a*). Oestrogen-related changes in sex hormone binding globulin levels during normal and gonadotrophin-stimulated menstrual cycles. *Clin. Endocrinol.*, **23**, 303.

DOWSETT, M., HARRIS, A.L., STUART-HARRIS, R. & 4 others (1985*b*). A comparison of the endocrine effects of low dose aminoglutethimide with and without hydrocortisone in post-menopausal breast cancer patients. *Br. J. Cancer*, **52**, 525.

FORBES, K.L., DOWSETT, M., ROSE, G.L., MUDGE, J.E. & JEFFCOATE, S.L. (1986). Dosage-related effects of danazol on sex hormone binding globulin and free and total androgen levels. *Clin. Endocrinol.*, **25**, 597.

GRODIN, J.M., SIITERI, P.K. & MACDONALD, P.C. (1973). Source of estrogen production in postmenopausal women. *J. Clin. Endocrinol. Metab.*, **36**, 207.

HARRIS, A.L., DOWSETT, M., JEFFCOATE, S.L., McKINNA, J.A., MORGAN, M. & SMITH, I.E. (1982). Endorcine and therapeutic effects of aminoglutethimide in premenopausal patients with breast cancer. *J. Clin. Endocrinol. Metab.*, **55**, 718.

HARRIS, A.L., DOWSETT, M., JEFFCOATE, S.L. & SMITH, I.E. (1983). Aminoglutethimide dose and hormone suppression in advanced breast cancer. *Eur. J. Cancer Clin. Oncol.*, **19**, 493.

HARRIS, A.L., DOWSETT, M., SMITH, I.E. & JEFFCOATE, S.L. (1984). Hydrocortisone alone vs. hydrocortisone plus aminoglutethimide: Comparison of the endocrine effects in postmenopausal breast cancer. *Eur. J. Cancer Clin. Oncol.*, **20**, 463.

JAFFE, R.B. (1986). The menopause and the perimenopausal period. In *Reproductive Endocrinology*, S.S.C. Yen & R.B. Jaffe, (eds) 2nd Edition, p. 406. Saunders: London.

JEPPSSON, S., GERSHAGEN, S., JOHANSSON, E.D.B. & RANNEVIK, G. (1982). Plasma levels of medroxyprogesterone acetate (MPA), sex hormone binding globulin, gonadal steroids, gonadotrophins and prolactin in women during long-term use of depo-MPA as a contraceptive agent. *Acta Endocrinol.*, **99**, 339.

MILLER, R.G. (1966). *Simultaneous statistical influence*. McGraw-Hill: New York, p. 15.

PANNUTI, F., MARTONI, A., LENAZ, G.R., PIANA, E. & NANNI, P. (1978). A possible new approach to the treatment of metastatic breast cancer: Massive dose of medroxyprogesterone acetate. *Cancer Treat. Rep.*, **62**, 499.

SAKAI, F., CHEIX, F., CLAVEL, M., COLON, J., MAYER, M., POMMATAU, E. & SAEZ, S. (1978). Increases in steroid binding globulins induced by tamoxifen in patients with carcinoma of the breast. *J. Endocrinol.*, **76**, 219.

SANTEN, R.J. (1982). In *Aminoglutethimide (Orimeten), Mechanism of Action and Clinical Results in Breast Cancer*, F.J.A. Paesi. (ed) p. 76. Ciba Geigy: Basel.

SIITERI, P.K., MURAI, J.T., HAMMOND, G.L., NISKER, J.A., RAYMOURE, W.J. & KUHN, R.W. (1982). The serum transport of steroid hormones. In *Recent Progress in Hormone Research*, R.O. Greep (ed) **38**, p. 457. Academic Press: New York.

STOLL, B.A. (1981). Breast cancer: Rationale for endocrine therapy. In *Hormonal Management of Endocrine-Related Cancer*, B.A. Stoll. (ed) p. 71. Lloyd-Luke: London.

STUART-HARRIS, R., DOWSETT, M., D'SOUZA, A. & 4 others (1985). Endocrine effects of low dose aminoglutethimide as an aromatase inhibitor in the treatment of breast cancer. *Clin. Endocrinol.*, **22**, 219.

SUFI, S.B., DONALDSON, A. & JEFFCOATE, S.L. (1986). *Method Manual of the Matched Assay Reagent Programme. WHO Special Programme of Research in Human Reproduction*. World Health Organisation, Geneva.

TSENG, L. & GURPIDE, E. (1975). Induction of human endometrial estradiol dehydrogenase by progestins. *Endocrinology*, **97**, 825.

VAN VEELEN, H., WILLEMSE, P.H.B., SLEIJFER, D.T., VAN DER PLOEG, E., SHUITER, W.J. & DOORENBOS, H. (1985*a*). Mechanism of adrenal suppression by high-dose medroxyprogesterone acetate in breast cancer patients. *Cancer Chemother. Pharmacol.*, **15**, 167.

VAN VEELEN, H., WILLEMSE, P.H.B., SLEIJFER, S.T., SHUITER, W.J. & DOORENBOS, H. (1985). Endocrine effects of medroxyprogesterone acetate: relation between plasma levels and suppression of adrenal steroids in patients with breast cancer. *Cancer Treat. Rep.*, **69**, 977.

VERMEULEN, A., VERDONCK, L., VAN DER STRAETEN, M. & ORIE, N. (1969). Capacity of the testosterone-binding globulin in human plasma and influence of specific binding of testosterone on its metabolic clearance rate. *J. Clin. Endocrinol.*, **29**, 1470.